# Reappraisal-related downregulation of amygdala BOLD activation occurs only during the late trial window

**DOI:** 10.3758/s13415-021-00980-z

**Published:** 2022-01-06

**Authors:** Jordan E. Pierce, R. James R. Blair, Kayla R. Clark, Maital Neta

**Affiliations:** 1 Cognitive and Affective Neuroscience Laboratory, Center for Brain, Biology, and Behavior, University of Nebraska-Lincoln, Lincoln, NE USA; 2 Center for Neurobehavioral Research, Boys Town National Research Hospital, Boys Town, NE USA

**Keywords:** Emotion regulation, Cognitive reappraisal, Amygdala, Prefrontal cortex, fMRI

## Abstract

During cognitive reappraisal, an individual reinterprets the meaning of an emotional stimulus to regulate the intensity of their emotional response. Prefrontal cortex activity has been found to support reappraisal and is putatively thought to downregulate the amygdala response to these stimuli. The timing of these regulation-related responses during the course of a trial, however, remains poorly understood. In the current fMRI study, participants were instructed to view or reappraise negative images and then rate how negative they felt following each image. The hemodynamic response function was estimated in 11 regions of interest for the entire time course of the trial including image viewing and rating. Notably, within the amygdala there was no evidence of downregulation in the early (picture viewing) window of the trial, only in the late (rating) window, which also correlated with a behavioral measure of reappraisal success. With respect to the prefrontal regions, some (e.g., inferior frontal gyrus) showed reappraisal-related activation in the early window, whereas others (e.g., middle frontal gyrus) showed increased activation primarily in the late window. These results highlight the temporal dynamics of different brain regions during emotion regulation and suggest that the amygdala response to negative images need not be immediately dampened to achieve successful cognitive reappraisal.

## Introduction

Emotion regulation allows an individual to define a goal state (e.g., to feel less sad), which can influence how emotional input is perceived and acted upon (Gross, 2015). One commonly studied regulation approach is cognitive reappraisal, which involves reinterpreting the meaning of a negative stimulus so that it is perceived as less unpleasant or salient (Buhle et al., 2014; Gross, 2015). Cognitive reappraisal is highly effective at reducing negative emotion both experimentally and clinically (Buhle et al., 2014; Gross & John, 2003; Ochsner et al., 2012). Many studies have investigated the mechanisms supporting this emotion regulation technique in healthy adults using functional magnetic resonance imaging (fMRI). Findings have consistently identified increased activation in the dorsolateral prefrontal cortex (PFC), ventrolateral PFC/inferior frontal gyrus (IFG), medial PFC/anterior cingulate cortex, posterior parietal cortex, and the lateral temporal lobe and, less consistently, decreased activation in the amygdala (Buhle et al., 2014; Frank et al., 2014; Kanske et al., 2011; Kohn et al., 2014; Ochsner et al., 2012; Petro et al., 2018).

One issue with studies of cognitive reappraisal is that often only a single metric of the BOLD response (peak amplitude) is considered, yet a trial occurs over an extended period (e.g., 4-30 seconds; Kalisch, 2009), so it is unclear what cognitive or affective processes during the trial recruit the relevant brain regions and how their responses may develop temporally. Indeed, the time course of the response is critically important to defining how the emotional reaction arises and is consciously experienced (Gross, 2015; Kalisch, 2009). According to the process model of emotion (Gross, 1998, 2015), reappraisal may engage cognitive/attention systems early to activate the desired regulation goals and shift focus to relevant stimulus features that promote reappraisal (Buhle et al., 2014; Ochsner et al., 2004; Suri et al., 2018). In one study that directly tested this prediction (Goldin et al., 2008), early activation was reported in medial PFC and left ventrolateral PFC/IFG that was associated with downregulation of a late response in the amygdala during a 15-second, disgust-inducing film clip. On the other hand, however, different regulation regions may be activated throughout the trial to maintain attention and monitor performance as emotional appraisals are repeatedly enacted and reassessed (Kalisch, 2009; Moors et al., 2013).

With respect to the decreased activation observed in the amygdala, this response is not always evident and may differ according to task design, analysis methods, or the regulation strategy used by participants (Dörfel et al., 2014; Kanske et al., 2011; McRae, Misra, et al., 2012b; Urry et al., 2006). For example, McRae, Misra, et al. (2012b) induced an emotional response through either a semantic (read a sentence) or perceptual (view a face) cue and found that cognitive reappraisal resulted in decreased amygdala activation only for the reading condition, with increased activation in the face condition. Additionally, Dörfel et al. (2014) specifically compared regulation strategies across subjects in a single study and found that while distraction, detachment, and expressive suppression resulted in decreased bilateral amygdala activation, reinterpretation (i.e., the type of reappraisal used in the current study) showed no overall activation difference from normal viewing.

Furthermore, when considering the time course of the amygdala response, two related studies examined its hemodynamic response function (HRF) during detachment-based regulation (Lamke et al., 2014; Walter et al., 2009) and found not only decreased left amygdala activation during regulation compared to viewing, but also decreased amygdala activation during a second passive viewing task, only for previously regulated images. This reduction in amygdala activation during the second viewing suggests that participants had successfully distanced themselves from the regulated images such that at a later time they no longer elicited an emotional response (Walter et al., 2009). However, in this prior study participants utilized a detachment strategy, which relies upon different processes than reappraisal to regulate emotion (Dörfel et al., 2014), and the time course of the amygdala response during cognitive reappraisal of negative images may differ.

In the current study, healthy participants completed a cognitive reappraisal task in which they were instructed to reinterpret negative images to make themselves feel less negative emotion, as reported in trial-by-trial ratings. We aimed to examine the HRF time course during the presentation of the emotional images and the subsequent rating period in regions previously associated with emotion regulation, including the amygdala and several locations within PFC (Buhle et al., 2014; Kanske et al., 2011; Ochsner et al., 2012). Specifically, to focus on areas that were most likely to be involved in the current reappraisal task, we constructed regions of interest from a previous study from our lab (Petro et al., 2018) using a similar task in which participants also were instructed to use reappraisal to downregulate negative emotional reactions to visual scenes. We expected to observe an increase in activation in dorsomedial PFC and IFG regions early in the reappraisal trials that corresponded to a relative decrease in amygdala activation later in the trial. In contrast, other attention- or cognitive control-related regions (e.g., lateral PFC) were expected to show sustained or increasing activation throughout reappraisal trials due to ongoing visual attention and working memory demands of the task. Finally, we predicted that individuals who exhibited a larger difference in amygdala activation for trials where they reappraised versus simply viewed negative images would show a larger corresponding reduction in behavioral ratings of experienced negative emotion.

## Methods

### Participants

As part of a larger study, 109 young adults were recruited from the community via publicly posted flyers for an initial behavioral session where they completed demographic forms and questionnaires related to emotion processing, and performed a valence judgement task on clear and ambiguous emotional images (that will not be described here). Inclusion criteria required participants to be between the ages of 17 and 60 years and right-handed. Exclusion criteria included previous history of a neurological or psychiatric disorder, including medication use for depression or anxiety, as well as any metal implants that were noncompatible with the MR environment (e.g., hair extension, braces, surgical implants). Of these participants, 91 were invited for a second session a week later and completed the MRI task described below. Ten participants data were excluded from analysis due to technical issues with recording behavioral responses in the MRI, and two additional participants were excluded based on a lack of task compliance (no behavioral responses for more than half of trials).

This resulted in a final sample size of 79 participants (40 females/39 males) with a mean age of 32.8 years (standard deviation [SD] = 11.3, range: 17-54), who reported their race as: 58 white/non-Hispanic (73.4%), 6 black (7.6%), 7 Asian (8.9%), 5 Hispanic/Latino (6.3%), 2 more than one race/Hispanic (2.5%), and 1 more than one race/non-Hispanic (1.3%). This sample size is consistent with the initial study goal that targeted data collection from 100 participants and is rather larger than much prior work (e.g., in the reappraisal meta-analysis of Buhle et al., 2014, the largest study had 42 participants). A post hoc power analysis in G*Power (Faul et al., 2007) indicated that with a sample size of 79, our *t*-tests could detect moderate effect sizes (d = 0.6) with power = 0.99 and smaller effect sizes (d = 0.3) with power = 0.75. All participants provided written, informed consent and received monetary compensation for their participation in the study; all research procedures were approved by the UNL Institutional Review Board.

### Task Design and Procedure

The emotion regulation task was designed based on a previously published event-related task (McRae, Gross, et al., 2012a). Each trial began with an instruction screen lasting 2 seconds with either “Look” or “Decrease” written on a green or blue background, respectively. This was followed by the presentation of an emotional image (from the International Affective Picture System (IAPS); Lang et al., 1997; see Appendix 1 for a list of selected items) for 7 seconds against the same colored background as the instruction screen. For the look instruction trials, half of the images were selected from those previously identified as having negative valence (“Look Negative”) and half of the images had neutral valence (“Look Neutral”); participants were instructed to respond naturally and allow whatever feelings may arise. For the decrease instruction trials (“Reappraise”), all the images were negative and participants were instructed to cognitively reinterpret the content to make themselves feel less negative, such as imagining that the image is from a movie or that assistance will arrive soon. Next, a rating screen appeared for 4 seconds where participants had to indicate the degree of negative emotion felt at the end of the image presentation (i.e., after reappraisal or viewing): “How bad do you feel?” on a scale from 1 (not at all) to 5 (very bad). Finally, there was a “Rest” screen that lasted 1, 2, or 3 seconds before the next trial began. While this brief rest period may not allow the BOLD response to return to baseline after each trial, by using a jittered rest period between different trial types, the HRF can be sufficiently estimated from the varying overlap of trial types and timings (Miezin et al., 2000). There were 20 trials each of Look Negative, Look Neutral, and Reappraise trials pseudo-randomly distributed throughout the task (60 total trials, all with unique images).

Participants were positioned on their back in the scanner and viewed the task screen via a mirror attached to the head coil. In the scanner just before beginning the emotion regulation task, participants were given detailed instructions and shown example images not used during the task itself (McRae, Gross, et al., 2012a). Participants then completed a set of three practice trials (2 “Decrease” and 1 “Look” trial) and afterwards were asked to explain how they reappraised each scene to ensure task comprehension. Specifically, the researcher ensured that the participant was reinterpreting the meaning of the image to make themself feel less bad by imagining a more positive context or outcome than their initial appraisal and not using another strategy, such as distraction that involved reduced attention to the image. Stimuli were presented using EPrime software (Psychological Software Tools, Inc., Pittsburgh, PA) and response ratings and reaction times were recorded via an MR-compatible button box. The anatomical scan was collected first, followed by two passive face viewing functional scans, the emotion regulation task, and finally a resting-state scan (only the regulation task will be described here). After the scan, participants completed a short debriefing interview to report their general adherence to the task, perceived success and difficulty, and broad reappraisal strategy as a final check for task compliance.

### MRI Acquisition Parameters

Scanning was performed at the Center for Brain, Biology, and Behavior (CB3) at UNL on a Siemens 3T Skyra scanner using a 32-channel head coil. Structural images were collected using a T1-weighted MPRAGE sequence with the following parameters: 192 interleaved slices, TR = 2.2 s, TE = 3.37 ms, voxel size = 1.0 mm^3^, matrix = 256 x 256, FOV = 256 mm^2^, flip angle = 7°. For the functional tasks, blood oxygen level-dependent (BOLD) activation was measured with an EPI sequence with the following parameters: 51 interleaved slices, multiband acceleration factor = 3, TR = 1.0 s, TE = 29.8 ms, voxel size = 2.5 mm^3^, matrix = 84 x 84, FOV = 210 mm^2^, flip angle = 60°, 474 volumes, total acquisition time = 8:08 per run. Slices were acquired parallel to the AC-PC plane.

### MRI Preprocessing and Statistical Analysis

Functional data were analyzed using the AFNI software package (Cox, 1996, 2012). Preprocessing included de-spiking of time series outliers, slice timing correction, alignment of functional volumes to each other and the individual anatomical image, standardization to the Talairach atlas space (Talairach & Tournoux, 1988), smoothing with a 6-mm FWHM kernel, and scaling of each voxel to a mean of 100. Next, the data were entered into a general linear model with regressors for each trial type (Reappraise, Look Negative, Look Neutral) using the “TENT” function to estimate the amplitude of the hemodynamic response at each TR from 0-16 seconds after stimulus onset (17 timepoints; TR = 1 second) without assuming a predetermined shape for the response in each voxel. Regressors of no interest included polynomials for each run (4 terms) and six motion parameters estimated during alignment (x, y, z shift/rotation). Look Neutral trials were included in the task to minimize habituation effects and as a general reference for emotional versus neutral responses but were not of primary interest in the current analysis.

**Fig. 1:**
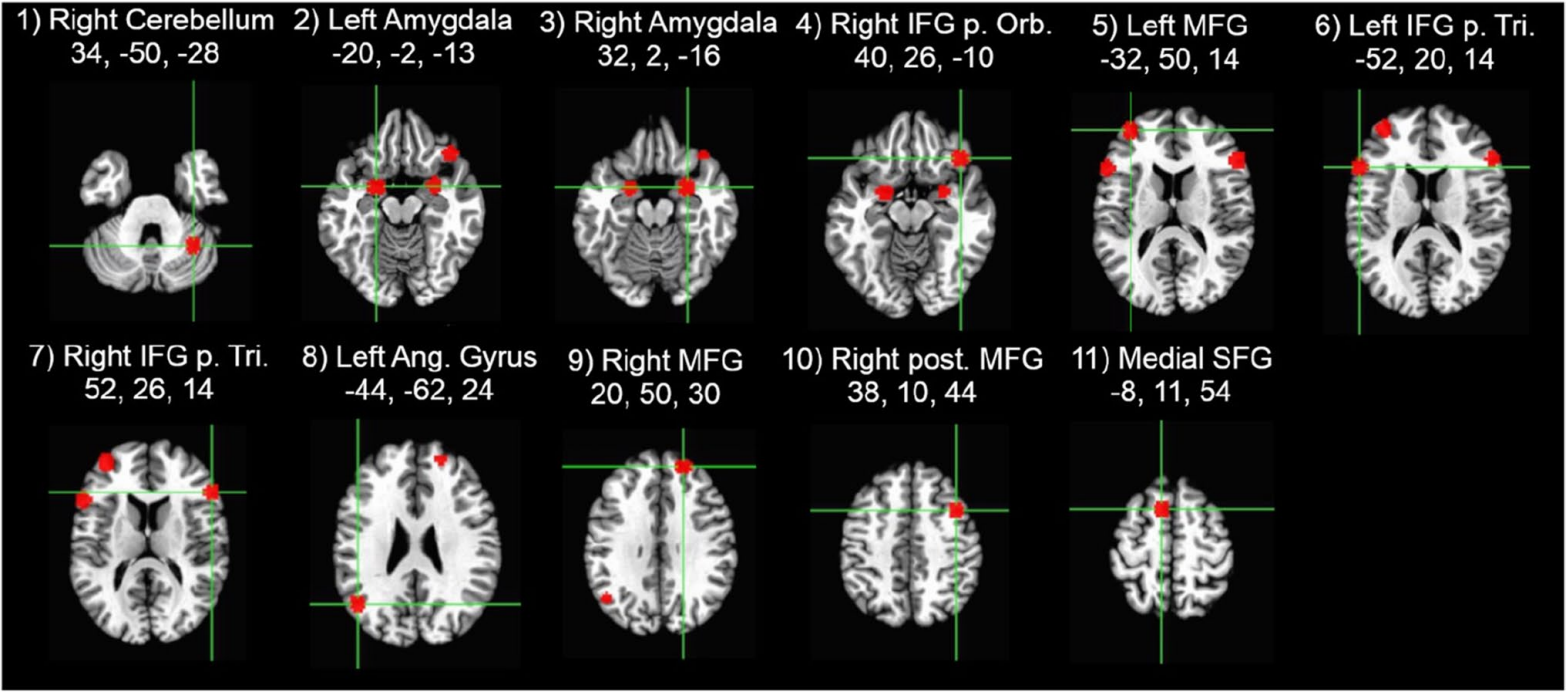
Regions of interest locations were defined based on Petro et al. (2018) and created with a 6-mm sphere centered on the peak coordinates (x,y,z), given in Talairach atlas space

The beta values for each time point (i.e., the estimated HRF) from each trial type then were extracted from 11 regions of interest (ROIs) based on a previous study of emotion regulation using reappraisal of negative images (Petro et al., 2018). Each ROI was defined as a 6-mm sphere centered on the peak coordinates for each location in their reappraise > maintain contrast (Fig. 1). To identify ROIs with similar temporal response patterns (Neta et al., 2015), the group average time courses for Reappraise and Look Negative trials were concatenated for each ROI separately (yielding an 11 x 34 matrix) and entered into a hierarchical clustering algorithm in Matlab (MathWorks, Natick, MA). The hierarchical tree was defined by using the unweighted paired group method with arithmetic mean (UPGMA), which calculates the mean distance between all pairs of data points in any two clusters and is best visualized using a dendrogram of the link distances. The number of clusters was selected by calculating the inconsistency coefficient for the height of each link compared with the average link height across the same level in the hierarchy, with the threshold value set to 1.

To compare the BOLD responses in early and late phases of the trial (putatively corresponding to the picture and rating periods), difference values (Reappraise-Look Negative) were calculated for each ROI and then averaged across time points from 4 to 8 seconds after picture onset (early window) and 11 to 15 seconds after picture onset (late window, i.e., 4 to 8 seconds after rating onset). These windows were selected to capture activation in the time around the potential peak responses following the onset of the picture or rating screen, given that the HRF typically peaks about 5-6 seconds after stimulus occurrence (Miezin et al., 2000). One sample *t*-tests (vs. 0) were conducted on the activation differences from the two time-windows in each ROI, with Holm’s adjusted *p*-values to account for multiple comparisons. Finally, the significant amygdala activation difference for Reappraise-Look Negative trials in the late window was correlated with the participants’ behavioral reappraisal success scores, which were calculated as the ratings for Look Negative trials minus the ratings for Reappraise trials multiplied by the Look Negative ratings (as in Petro et al., 2018). To explore possible behavioral correlations with activity in other regions, the correlations between other ROIs with significant differences in either the early or late window and behavioral reappraisal success were also analyzed. Correlations were calculated with Pearson’s correlation coefficient and significance levels were set to *p* < 0.05. Statistical analyses were performed using JASP software (JASP Team (2020), version 0.12.2 [computer software]).

## Results

### Behavior

Participants’ behavioral ratings were higher (i.e., a more negative feeling; 1 to 5 scale) for Look Negative trials (mean (SD) = 3.79 (0.49)) compared with Reappraise trials (2.58 (0.55); *t*(78) = 15.9, *p* < 0.001), and both Look Negative (*t*(78) = 32.1, *p* < 0.001) and Reappraise (*t*(78) = 16.2, *p* < 0.001) trials were rated higher than Look Neutral trials (1.36 (0.49)). For reaction times, responses were slower for Reappraise trials (1,530 ms (399)) compared with both Look Negative (1,248 ms (338); *t*(78) = 9.2, *p* < 0.001) and Look Neutral (1,008 ms (308); *t*(78) = 17.0, *p* < 0.001) trials, which also differed from each other (*t*(78) = 7.8, *p* < 0.001). The mean reappraisal success score (which was calculated as (Look Negative-Reappraise rating)*Look Negative rating, with larger positive values indicating a greater reduction in negative feeling on Reappraise trials) was 4.74 (SD = 2.90, range: −1.35 to 13.86). A 3x2 ANOVA (trial type by sex) was conducted on behavioral measures to compare performance between male and female participants. There was a significant effect of sex for reaction times (*F*(1,77) = 4.40, *p* = 0.039), such that males responded more slowly overall (1,335 ms) than females (1,190 ms). The interaction between trial type and sex was not significant for reaction times, and there were no sex effects on trial ratings. An independent *t*-test on reappraisal success also indicated no significant sex differences. Finally, due to the wide age range in our adult sample, age effects on reappraisal also were considered. Age was correlated with reappraisal success (*r* = −0.305, *p* = 0.006), such that younger adult participants exhibited greater reappraisal success than older participants.

### BOLD Responses

**Fig. 2:**
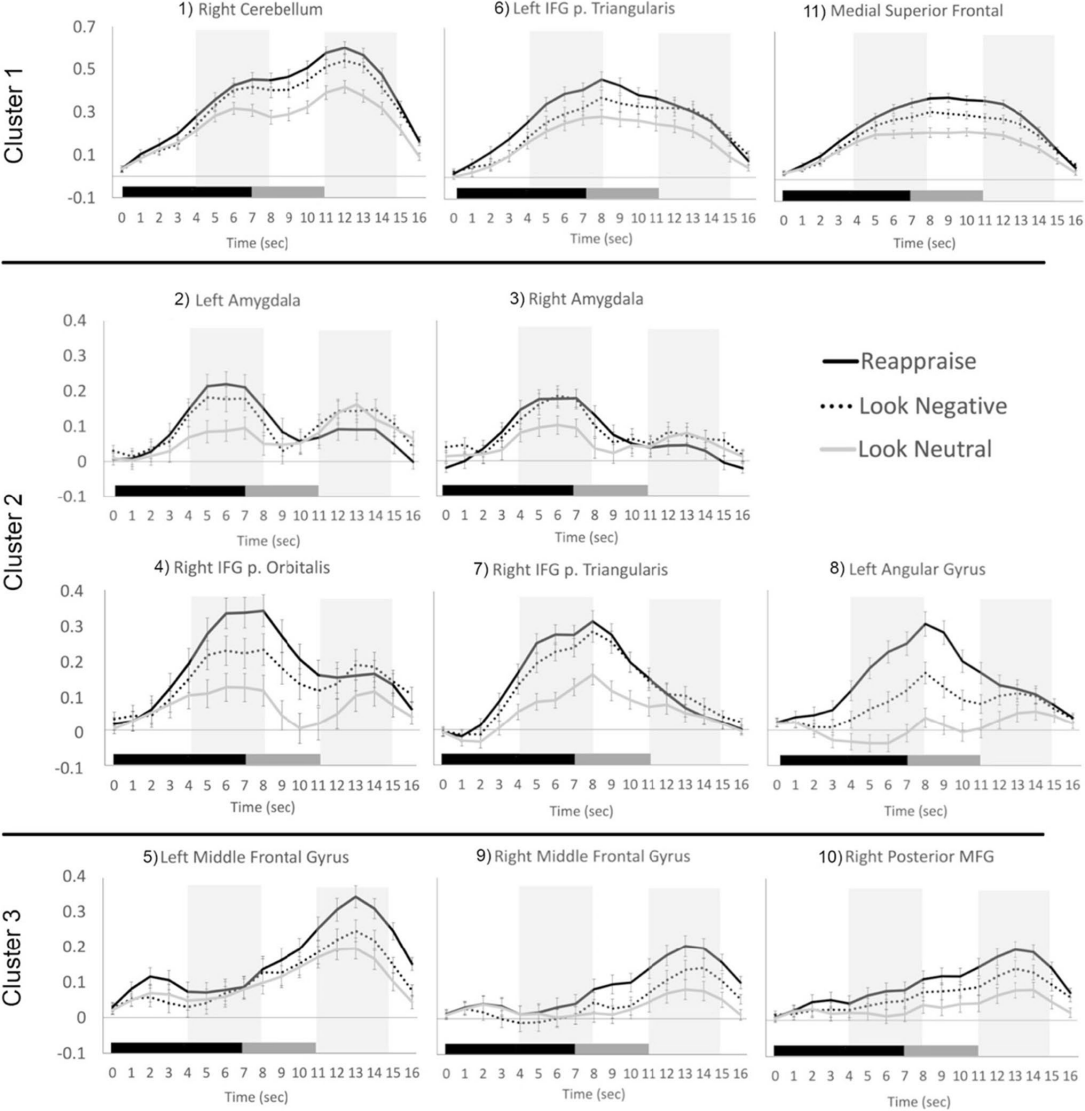
Hemodynamic response functions for Reappraise (black), Look Negative (dashed), and Look Neutral (gray) trials in each of the 11 ROIs. ROIs are grouped into three clusters based on the concatenated time courses for Reappraise and Look Negative trials only. The stimulus picture appeared at time 0 and lasted 7 seconds (black bar), then the rating screen appeared and lasted 4 seconds (gray bar), followed by 1-3 seconds of rest. Shaded areas show early and late windows. Errors bars show ±1 SE

**Fig. 3:**
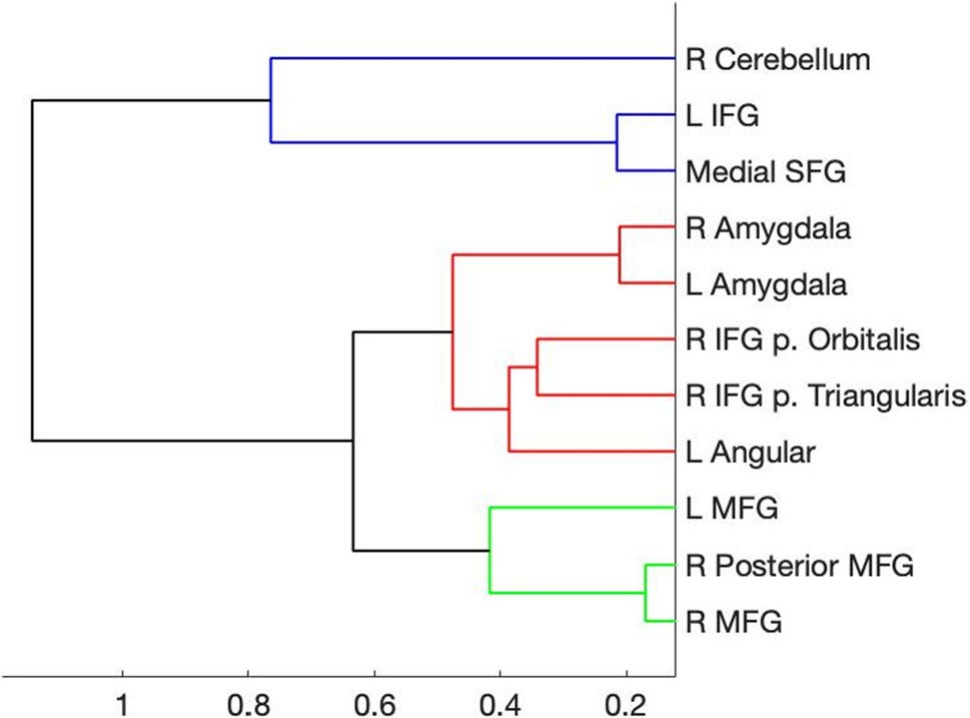
Hierarchical clustering dendrogram. Clustering results were based on concatenated Reappraise and Look Negative time courses. Colors represent the three identified clusters with the most similar time courses, as shown in Fig. 2

Figure 2 illustrates the estimated HRF time courses for each of the three trial types in each of the 11 ROIs. Descriptively, as expected, the trials with negative stimuli elicited stronger responses than the neutral trials, but the Reappraise trials showed even stronger peak responses than Look Negative trials in most of these emotion regulation regions. Using a hierarchical clustering algorithm, the Reappraise and Look Negative trial HRFs from each ROI then were grouped into three clusters with similar time course patterns (Fig. 3). The first cluster included the right cerebellum, left IFG pars triangularis, and medial superior frontal gyrus (SFG; ROIs #1, 6, and 11 in Fig. 1), and the time courses generally showed strong activation throughout the whole trial. The second cluster included bilateral amygdala, right IFG pars orbitalis and pars triangularis, and left angular gyrus (ROIs #2, 3, 4, 7, and 8) and showed a stronger response early in the trial, which tended to decrease in the second half of the trial. The third cluster included the bilateral MFG and right posterior MFG (ROIs #5, 9, and 10) and showed a weak response early in the trial, which increased in the second half of the trial.

**Fig. 4:**
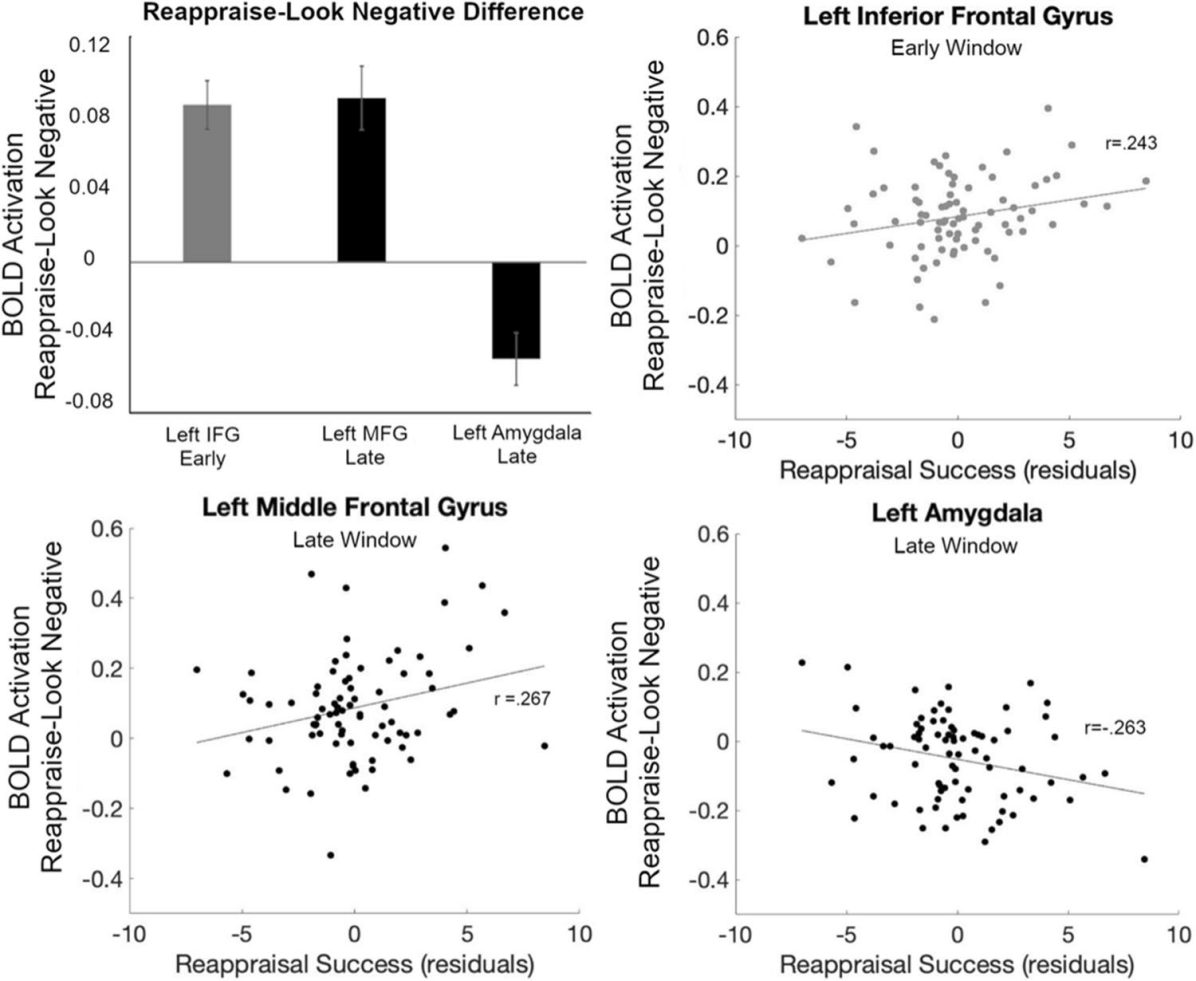
Brain-behavior correlations. Average Reappraise-Look Negative BOLD signal difference in the early or late windows for the three ROIs with significant behavioral correlations (*upper left*). Correlations between reappraisal success (controlling for age) and the BOLD signal were significant for the left IFG (ROI #6; *upper right*) during the early window and left MFG (ROI #5; *lower left*) and left amygdala (ROI #2; *lower right*) during the late window

To quantify the differences between Reappraise and Look Negative trials, BOLD response values from early (4 to 8 seconds after picture onset) and late (11 to 15 seconds after picture onset) time windows were averaged in each ROI and compared with zero (means and statistics are listed in Table 1). Notably, the bilateral amygdala showed a signficant *decrease* only in the late (but not early) time window, whereas the left MFG showed a significant *increase* only in the late window. Finally, to investigate whether the amygdala response was related to behavioral reappraisal success, correlations were examined between the activation difference between Reappraise and Look Negative trials in the late window and behavior. Reappraisal success (controlling for the effect of age) was negatively correlated in the late window with the left amygdala BOLD signal difference (*r* = −0.263, *p* = 0.020; Fig. 4). To explore other regions’ relationship with behavior, the correlations between ROIs with significant differences in either the early or late window and behavioral reappraisal success also were analyzed. Consistent with our predictions, reappraisal success was positively correlated in the early window with left IFG (*r* = 0.243, *p* = 0.032) and in the late window with left MFG (*r* = 0.267, *p* = 0.018); no other ROIs or time windows had significant correlations.

**Table 1:** Results from one-sample *t*-tests on Reappraise minus Look Negative activation in the early (4 to 8 seconds after picture onset) and late (11 to 15 seconds after picture onset) time windows. *P* values are corrected for multiple comparisons using Holm’s adjustment. Effect sizes are given as Cohen’s *d*

Region	Mean (SD)	*t*	Holm’s *p*	Cohen’s *d*
Early window				
1) Right cerebellum	**0.034 (0.095)**	**3.201**	**0.014**	**0.360**
2) Left amygdala	0.032 (0.145)	1.960	0.162	0.221
3) Right amygdala	0.014 (0.128)	0.941	0.349	0.106
4) Right IFG p. orbitalis	**0.086 (0.146)**	**5.252**	**0.011**	**0.591**
5) Left MFG	0.019 (0.128)	1.286	0.404	0.145
6) Left IFG p. triangularis	**0.084 (0.115)**	**6.456**	**0.011**	**0.726**
7) Right IFG p. triangularis	**0.041 (0.111)**	**3.283**	**0.014**	**0.369**
8) Left angular gyrus	**0.122 (0.110)**	**9.805**	**0.011**	**1.103**
9) Right MFG	**0.030 (0.100)**	**2.672**	**0.045**	**0.301**
10) Right posterior MFG	**0.028 (0.096)**	**2.593**	**0.045**	**0.292**
11) Medial SFG	**0.051 (0.074)**	**6.062**	**0.011**	**0.682**
Late window				
1) Right cerebellum	**0.051 (0.121)**	**3.775**	**0.011**	**0.425**
2) Left amygdala	**−0.051 (0.124)**	**−3.681**	**0.011**	**-0.414**
3) Right amygdala	−0.035 (0.131)	−2.347	0.105	-0.264
4) Right IFG p. orbitalis	0.000 (0.166)	0.018	1.00	0.002
5) Left MFG	**0.087 (0.151)**	**5.160**	**0.011**	**0.581**
6) Left IFG p. triangularis	0.006 (0.124)	0.467	1.00	0.052
7) Right IFG p. triangularis	−0.014 (0.121)	−1.054	0.885	-0.119
8) Left angular gyrus	0.029 (0.127)	2.020	0.188	0.227
9) Right MFG	**0.063 (0.103)**	**5.424**	**0.011**	**0.610**
10) Right posterior MFG	**0.052 (0.100)**	**4.727**	**0.011**	**0.532**
11) Medial SFG	**0.044 (0.090)**	**4.363**	**0.011**	**0.491**

## Discussion

In this study, the time courses of the BOLD signal in brain regions supporting emotion regulation were examined while participants used cognitive reappraisal to regulate their emotional response to negative images. The HRF time courses were extracted from 11 ROIs previously associated with a similar reappraisal task (Petro et al., 2018) and analyzed in early and late time windows corresponding roughly to the picture viewing and emotion rating periods of the trial, respectively. Interestingly, the bilateral amygdala did not show a significant difference in activation for Reappraise compared with Look Negative trials in the early (picture) window but did show a significant decrease in the late (rating) window. Additionally, regions of the PFC differed in their response patterns; some regions (e.g., IFG) exhibited a relative increase for reappraisal only in the early window, others (i.e., bilateral MFG) showed a significant increase primarily in the late window, and still others (e.g., medial SFG) showed an increase throughout the entire trial. Notably, the decreased activation in left amygdala in the late window was correlated with reappraisal success across participants, demonstrating that this late response (but not the early reactivity during active reappraisal) was reflective of an individual’s ultimate ability to downregulate their emotional response.

### Decreased Amygdala Response only in the Late Window

When examining the time course of the BOLD response, amygdala activation in the early (picture viewing) window was not significantly different between Reappraise and Look Negative trials, but amygdala activation in the late (rating) window was decreased for Reappraise compared with Look Negative trials. Although some previous studies have found decreased amygdala activation during reappraisal (Ochsner et al., 2004), the largest reductions in amygdala activation have been found in tasks that employ distraction or distancing approaches to emotion regulation (see also Dörfel et al., 2014; McRae et al., 2010). Prior work that did not report a reappraisal-related attenuation of amygdala activity may reflect the response only from the early phase of reappraisal, as shown in the current findings. We speculate, on the basis of arguments presented in earlier work (McRae et al., 2010), that reinterpretation strategies may require further engagement with the emotional image and processing of visual affective cues (e.g., searching for a plausible interpretation that is less negative). This may prohibit a strong early amygdala attenuation, at least until a new interpretation is finalized.

Subsequently, when the participant rates the negativity of the image in the late trial window, the successfully reappraised images generate a weaker amygdala response (cf. decreased amygdala response during second passive viewing of previously regulated images in Walter et al., 2009; see also Denny et al., 2015). Indeed, reappraisal-related amygdala activation in the late window was related to behavioral reappraisal success: participants showing a greater reduction in left amygdala activity during the rating period also showed a greater reduction in their negative rating for Reappraise compared with Look Negative trials. Furthermore, there was a negative correlation between reappraisal success and age within this adult sample: younger participants showed greater reappraisal success (i.e., a greater relative reduction in Reappraise negative ratings) than older participants. Younger adults, therefore, may have had better reappraisal ability (Opitz et al., 2012; Tucker et al., 2012) or greater cognitive flexibility than older adults (Fisk & Sharp, 2004; Hedden & Gabrieli, 2004) or, perhaps, were more likely to exaggerate how well they reduced their negative feelings to meet perceived researcher expectations. Ultimately, the relationship between decreased amygdala activation and behavior suggests that modeling the amygdala response over time can provide a better, objective neural marker of reappraisal success that is related to behavior, yet not susceptible to demand characteristics (as behavioral measures of reappraisal success may be).

### Variable Response Dynamics in Regions of PFC

Beyond the amygdala, other emotion regulation regions including those in PFC showed an increase in activation during reappraisal compared to looking naturally at the negative images. This is consistent with the expected involvement of these regions based on the prior study (requiring reappraisal of the same negative images) from which the ROIs were taken (Petro et al., 2018), as well as meta-analyses of emotion regulation (Buhle et al., 2014). In this study, however, the temporal dynamics of the responses in these regions were of particular interest. The analysis of the time course differences revealed a strong response on Reappraise trials in IFG and medial SFG during the early window. This finding supports previous studies showing that cognitive reappraisal can recruit portions of PFC early in the trial before the initial emotional response to the stimulus fully develops, in line with the process model of emotion (Goldin et al., 2008; Gross, 2015), which was found here using a larger sample that included both men and women and stimuli that elicited other emotions like fear rather than only disgust (compared with Goldin et al., 2008). The IFG’s involvement early in the trial suggests a role in selection and inhibition of emotional appraisals, consistent with previous reports on the function of inferior and ventrolateral PFC (Aron et al., 2014; Buhle et al., 2014; Papousek et al., 2017). The strong response in both the early and late window in medial SFG, on the other hand, may reflect an extended attention maintenance or monitoring role in reappraisal for this region. Finally, it is worth noting that the right cerebellum also showed strong, increased activation for reappraisal throughout the trial. This finding may be related to the use of a motor response for the ratings, yet the specific increase for Reappraise trials suggests that this region might be cooperating with PFC in a more cognitive manner, such as tuning attention to emotion appraisals, in line with recent reports of broader cerebellar involvement in cognition and emotion (Buckner et al., 2011; Pierce & Péron, 2020; Schmahmann, 2019).

In addition to the early effect in some regions of PFC, the bilateral anterior MFG had significantly greater activation for Reappraise compared to Look Negative trials only in the late window, following the rating period. This region of PFC, therefore, may have been less involved in early regulation processes described above and more involved in later evaluation of the participant’s subjective experience based on the new appraisal. Conversely, this area of MFG may indeed participate in reappraisal of the emotional images, but this may not occur until later in the trial when a behavioral response (rating) must be made. Moreover, given the inverse pattern of activity in the amygdala and MFG in the late window, this region also may be contributing to the late downregulation of the amygdala response (possibly via ventromedial PFC, which has direct anatomical connections to the amygdala; Ghashghaei et al., 2007; Silvers et al., 2017). This is further supported by the positive correlation between left MFG activation and a reduction in negative feeling ratings, mirroring the effect in left amygdala. The late effect in this PFC region, however, may be overlooked in studies that use shorter trials or do not consider the temporal evolution of the reappraisal response (Gonzalez-Castillo et al., 2012; Neta et al., 2015).

### Lateralization of Reappraisal Effects

In addition to the temporal differences described above, the current results also exhibited a lateralization effect wherein each of the significant brain-behavior correlations occurred in a left hemisphere ROI (amygdala, MFG and IFG), a pattern that has been reported in some, but not all, previous studies of reappraisal. For example, greater left frontal activity has been reported in association with a decreased negative emotional response during reappraisal (Choi et al., 2016) and the ability to generate alternate appraisals of a stimulus (Papousek et al., 2017). Additionally, disrupted left PFC activity may lead to worse reappraisal performance in aging populations (Opitz et al., 2012) or those with mood disorders (Johnstone et al., 2007). The proposed frontal asymmetry is further supported by a meta-analysis of early fMRI reappraisal studies (Kalisch, 2009), which reported a shift in peak activation from left to right PFC as the duration of the reappraisal period increased from 4 to 26 seconds. The author suggested that this difference may arise due to a cognitive shift from the implementation of new appraisals to maintenance processes over the course of a reappraisal trial. In the current study, the left lateralized effects were not limited to the early window, however, meaning that this lateralized PFC activity may be driven by a range of cognitive functions, such as the inhibition of initial emotional appraisals in IFG or language demands of generating new appraisals in left lateral PFC (Dörfel et al., 2014; Ochsner et al., 2012). Nonetheless, activations (that did not correlate with behavioral reappraisal) were also identified bilaterally in PFC, indicating that while there was some evidence of asymmetry in frontal responses during reappraisal, both hemispheres were recruited by the current task.

### Limitations

One methodological limitation that must be considered is that the HRFs were extracted from a 16-second time window that encompassed both the image presentation itself and the subsequent rating screen, which always immediately followed the image. Therefore, it is not possible to determine the extent to which the late responses were driven by slow reactions to the picture, the offset of the picture, or the rating screen itself (Goldin et al., 2008; Lamke et al., 2014; Walter et al., 2009). Indeed, in several ROIs the HRF was elevated across the whole trial with the response to the picture and the rating being relatively indistinguishable. Conversely, some regions, such as, crucially, the amygdala, did have distinct response peaks in the early and/or late windows. Furthermore, in the current study, participants were instructed specifically to cognitively reinterpret the images and not to simply distance themselves from the negative stimuli (or use some other form of reappraisal or distraction). While this distinction was confirmed during practice and in post-scan debriefing, it is difficult to guarantee that all participants implemented reappraisal appropriately on each trial. Future work that examines the time course of reinterpretation and distancing (or other strategies) within participants could offer more elaborated training on different strategies to ensure that participants can identify clearly which approach they actually used for a given trial and how the amygdala responds to each strategy over time.

## Conclusions

This work lends new insight into the mechanisms that support cognitive reappraisal as a strategy for downregulation of negative emotions during a trial in which participants were asked to either view an image naturally or reinterpret it to feel less negative. We investigated the BOLD time course from regions involved in reappraisal, including the amygdala and various areas of the PFC. Importantly, this approach was crucial for identifying the different response patterns in emotion regulation regions that may contribute to different inhibitory, attentional, and/or evaluative processes during different periods of a reappraisal trial and offers one possible explanation of the inconsistent findings in the literature related to downregulation of amygdala. Future research that manipulates the timing of reappraisal image viewing and rating periods or the regulation strategy implemented could target these effects directly to clarify the characteristics of the temporal response in the amygdala and PFC.

## Data Availability

Data are available from the corresponding author upon request. None of the experiments were preregistered.

## Appendix 1 IAPS images used for negative and neutral trial stimuli.

**Table:**

Negative	17; 2141; 2205; 2683; 2800; 3160; 3180; 3220; 3230; 34; 3530; 3550; 37; 43; 6210; 6212; 6250; 6300; 6312; 6370; 6550; 6570.1; 6831; 8230; 9050; 9181; 9320; 9400; 9420; 9421; 9425; 9430; 9470; 9490; 9520; 9571; 9600; 9620; 9910; 9921
Neutral	2190; 2200; 2440; 2441; 2493; 2516; 7002; 7004; 7006; 7009; 7025; 7040; 7050; 7090; 7100; 7175; 7211; 7233; 7235; 7950
